# A Case of *Pseudomonas straminea* Blood Stream Infection in an Elderly Woman with Cellulitis

**DOI:** 10.3390/idr16040053

**Published:** 2024-07-29

**Authors:** Leopold Böhm, Marius Eberhardt Schaller, Carsten Balczun, Andreas Krüger, Timo Schummel, Alexander Ammon, Niklas Klein, Dario Lucas Helbing, Rüdiger Eming, Frieder Fuchs

**Affiliations:** 1 Department of Microbiology and Hospital Hygiene, Bundeswehr Central Hospital Koblenz, 56072 Koblenz, Germany; leopoldboehm@bundeswehr.org (L.B.); carsten1balczun@bundeswehr.org (C.B.); andreas7krueger@bundeswehr.org (A.K.); timoschummel@bundeswehr.org (T.S.); niklasklein@bundeswehr.org (N.K.); 2 Department of Dermatology, Venerology and Allergology, Bundeswehr Central Hospital Koblenz, 56072 Koblenz, Germany; mariusschaller@bundeswehr.org (M.E.S.); ruedigereming@bundeswehr.org (R.E.); 3 Department of Pathology, Bundeswehr Central Hospital Koblenz, 56072 Koblenz, Germany; alexanderammon@bundeswehr.org; 4 Department of Psychiatry and Psychotherapy, Jena University Hospital, Friedrich Schiller University Jena, Philosophenweg 3, 07743 Jena, Germany; dario.helbing@leibniz-fli.de; 5 Leibniz Institute on Aging, Fritz Lipmann Institute, 07745 Jena, Germany; 6 Institute for Medical Microbiology, Immunology and Hygiene, University of Cologne, Medical Faculty and University Hospital of Cologne, 50931 Cologne, Germany

**Keywords:** Bacteremia, BSI, chronic diseases, psoriasis, atopic dermatitis, cellulitis, skin ulcer, *Galleria mellonella*, *P. aeruginosa*

## Abstract

Here, we report the simultaneous isolation of *Pseudomonas straminea* from blood cultures and from a skin ulcer in an elderly woman who suffered from atopic dermatitis and psoriasis and developed acute cellulitis of both arms requiring hospital treatment. To the best of our knowledge, *P. straminea* has not been previously reported to cause invasive infections in humans. This case highlights how chronic diseases and older age increase the susceptibility to bacterial infections with environmental bacteria of low virulence. Our study describes the microbiological identification of the blood culture isolate, including morpho-molecular characterization and virulence demonstration in a *Galleria mellonella* model.

## 1. Introduction

*Pseudomonas* spp. is a genus of widespread, environmental bacteria that can be found in different habitats such as plants, water, and soil. Except for *Pseudomonas aeruginosa*, representatives of other *Pseudomonas* spp. do not commonly cause invasive diseases in humans. Recovery from clinical specimens is therefore rare for *Pseudomonas* spp. other than *P. aeruginosa*; however, some species such as *Pseudomonas putida* or *Pseudomonas fluorescens* have been reported to cause human infection [1,2]. Due to the diverse intrinsic mechanisms contributing to antimicrobial resistance, *Pseudomonas* spp. can cause difficult-to-treat infections. Like other *Pseudomonas* spp., *Pseudomonas straminea* is a Gram-negative rod-shaped aerobic and motile bacterium [3]. First isolated from Japanese rice plantations, it was formerly named *Pseudomonas ochracea*. *P. straminea* represents an orthographic variant that belongs, among other species (e.g., *Pseudomonas mendocina* or *Pseudomonas alcaligenes*), to the *P. aeruginosa* group based on similarities in the 16s rDNA sequence [4]. It is also phylogenetically adjacent to the plant bacteria *Pseudomonas flavescens* [5,6] and *Pseudomonas argentinensis* [7]. A recent study proposed a *P. straminea* clade based on these similarities [8]. As of today, the isolation of *P. straminea* from clinical specimens or any association with infectious diseases in humans has, to the best of our knowledge, not been reported.

## 2. Case Presentation

An 82-year-old female patient from Germany sought care at the emergency department (ED) of the Bundeswehr Central Hospital Koblenz. She presented redness affecting the entire left arm, associated with a progressively painful sensation and general fatigue, and an ulcer on the back of her right hand. The patient had received prior care from a phlebologist during an elective consultation, which was scheduled as a regular check-up for her chronic venous insufficiency affecting both the upper and lower extremities. During the visit, the phlebologist considered a bacterial infection on her left arm and immediately arranged presentation at the ED.

**Figure 1:**
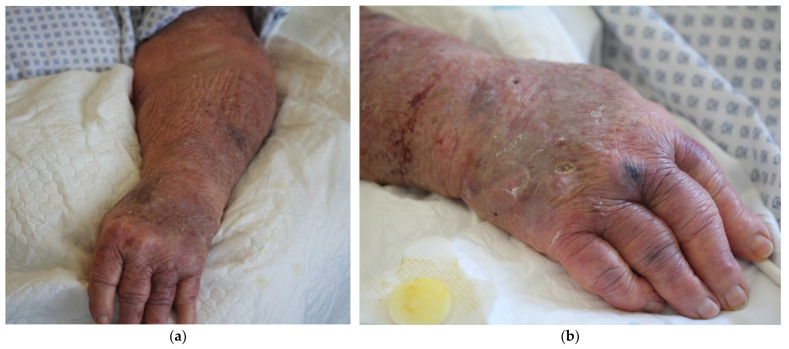
Clinical presentation of the patient with cellulitis affecting the entire left arm and an ulcer on the dorsum of the right hand: (**a**) Sharply demarcated, dark, in places livid, erythema with doughy edematous swelling over the entire left arm. Overall marked poikilodermic skin appearance and multiple scarring residuals, hematomas, and marked skin atrophy; (**b**) ulceration measuring approximately 0.5 cm × 1 cm on the right dorsum of the hand, proximal to the IV metacarpophalangeal joint, with a whitish, slightly hyperkeratotic, flocculently destructed wound bed. The ulceration was the sampling site of the bacterial swab that grew *P. straminea*. Multiple suggillations are visible on the extensor sides of the fingers.

The examination in the ED revealed an edematous swelling affecting both forearms, a sharply demarcated erythema affecting the entire left arm, and an isolated crusty ulceration on the right back of the hand (Figure 1). Areas affected by the erythema also presented with overheating and allodynia. The patient reported that the ulceration stemmed from lichen ruber planus and noted that she constantly suffered from one or two wounds that would take from weeks to months to heal. She also reported suffering from several other chronic diseases of the skin including atopic dermatitis, psoriasis, and different allergies, including allergies to penicillin, preservative agents, and bee stings. Although she had received several courses of glucocorticoid and methotrexate treatment in the past, she only received apremilast, a selective inhibitor inhibiting phosphodiesterase 4, for her psoriatic arthritis as an immunomodulatory medication at the time of admission. The symptoms were diagnosed as cellulitis. The patient was admitted and treatment with antiseptic skin poultices (octenisept^®^) and clindamycin (clindamycin, 1800 mg/day, 1-1-1) was initiated. 

Clindamycin was applied intravenously through a newly implanted central venous catheter (CVC) and blood cultures were taken from the freshly implanted CVC. Peripheral venous puncture could not be applied to any veins of the extremities because all potential puncture sites were affected by either swelling, erythema, overheating, allodynia, or ulcerations or a combination of these symptoms. After two days of inpatient treatment with clindamycin, increasing levels of laboratory infection parameters were recognized (increase in c-reactive protein (15.76 mg/dL to 29.89 mg/dL), procalcitonin (0.22 ng/mL to 0.62 ng/mL), and interleukin 6 (287 pg/mL to 2502 pg/mL)). Because of these increases and the clinical persistence of the cellulitis, the antibiotic treatment was adjusted to also target Gram-negative pathogens. Since the patient reported a not well-defined allergy to penicillin, cefotaxime was initiated to avoid even unlikely risks of allergic complications. Soon after switching to cefotaxime, *P. straminea* was isolated from one aerobic blood culture from the CVC implantation and from a bacterial swab from the ulcer on the dorsum of the right hand (Figure 1). The accompanying anaerobic bottles remained sterile. The antibiotic regimen was changed to ceftazidime (6 g/day, 1-1-1) as cefotaxime is generally not recommended to treat infections with *Pseudomonas* spp. [9]. The isolate was reported as susceptible to ceftazidime (Table 1, susceptible, increased exposure) based on the EUCAST breakpoints for *Pseudomonas* spp. (Version 13.00, valid from 1 January 2023). From the wound swab, *Enterococcus faecalis,* in addition to *P. straminea,* was also isolated. The change in the antibiotic regimen led to a rapid clinical recovery of the patient. Ceftazidime treatment was applied for a total of 6 days until a full recovery from the bacterial infection was seen, monitored by inpatient visits and laboratory values. Also, the CVC was replaced after the isolation of *P. straminea.* After a day of ceftazidime treatment, two pairs of follow-up blood cultures were drawn to rule out the persistence of the germ. However, all follow-up cultures from the wound and CVC remained sterile for the total incubation time, and the systemic and local symptoms caused by the bacterial infection showed resolution. The hospital stay was prolonged due to an electrolyte imbalance that required treatment for which the patient was transferred to another department and discharged from the hospital after a total inpatient stay of 14 days.

## 3. Materials and Methods

**Figure 2:**
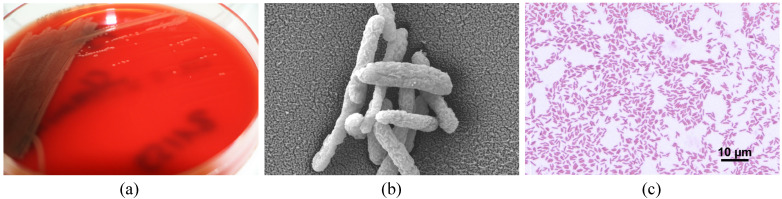
Colony morphology and microscopic morphology of *P. straminea*: (**a**) Culture growth of *P. straminea* on Columbia blood agar. Colonies appear small and round with a dull, greyish-yellow color; (**b**) scatter electron microscopy image of cultured *P. straminea* colonies with 26.02 K resolution showing slightly curved or straight rods with band-shaped structures that can be assessed as shrinkage artifacts; (**c**) transmitted light microscopy image of a Gram stain of a smear from cultured *P. straminea* colonies showed scattered, Gram-negative rods with an average length of 1.3–1.8 μm.

*P. straminea* grew on standard media including Columbia blood agar, Chocolate agar, and MacConkey agar (BD GmbH, Heidelberg, Germany) under aerobic conditions at 35 ± 1 °C. Colonies appeared smooth, round, and raised with a dull, greyish-yellow color and without hemolysis on Columbia blood agar (Figure 2). The colonies were small with an average diameter of 550 to 700 μm. In contrast to *P. aeruginosa*, colonies of *P. straminea* did not elicit a distinct smell. *P. straminea* was non-fermentative and catalase- and oxidase-positive. The Gram stain and scatter electron microscopy of cultured *P. straminea* samples showed scattered, slim, slightly curved or straight, Gram-negative rods with an average length of 1.3–1.8 μm (Figure 2). 

### 3.1. Antimicrobial Susceptibility Testing

Antibiotic susceptibility was tested using the VITEK^®^ 2 System (Biomerieux, Nürtingen, Germany) using the AST-N429 card type based on the EUCAST breakpoints for *Pseudomonas* spp. (Version 13.00, valid from 1 January 2023).

### 3.2. Identification

Matrix-assisted laser desorption ionization–time of flight mass spectrometry (MALDI-TOF MS) with a MALDI Biotyper^®^ (Bruker Corporation, Bremen, Germany) was applied and suggested *P. straminea* with a consistency rating of 2.09 (MBT reference library version 12.0.0.0), corresponding to consistency on the species level. Biochemical identification was conducted with the VITEK^®^ 2 System (Biomerieux, Nürtingen, Germany) using an identification card (VITEK II GN ID card type), yet with this method, low discrimination without any suggestion to species- or genus-ID was reported. Outside the clinical routine, PCR amplification of the 16S rDNA locus using conserved primers and subsequent sequencing using BLAST search against NCBI GenBank identified a 99.1% identity to *P. straminea* entries (accession numbers LC507441, LC507440, and LC420056) (see Section 3.3 for details). 

### 3.3. Molecular Characterization and Accession to Data

A colony of the blood culture isolate of *P. straminea* was transferred into a 1.5 mL reaction tube containing 180 μL of ATL buffer and the sample was mixed by vigorous stirring. DNA from this sample was isolated using the QIAamp DNA Mini and BloodMini Kit according to the manufacturer’s instructions (Qiagen, Hilden, Germany): Proteinase K (20 μL) was added and the sample was incubated at 56 °C and occasionally vortexed until completely lysed. After adding 200 μL of buffer AL, the sample was incubated at 70 °C for 10 min. Following these lysis steps, the sample was mixed with 200 μL of ethanol (100% *v*/*v*) and then processed using a QIAamp Mini spin column as described in the kit’s handbook for DNA purification from tissues. The DNA was eluted in 100 μL of nuclease-free water. PCR for amplification of 16S rDNA using primers 16S27F and 16S1492R was carried out in a total volume of 50 μL with a 0.2 μM final concentration of each primer and 1 μL of isolated DNA using the Taq PCR Core Kit (Qiagen) as recommended by the manufacturer. DNA was amplified using an initial step of 94 °C for 3 min and 40 cycles of 94 °C for 1 min, 52 °C for 1 min, and 72 °C for 3 min, followed by a final step of 72 °C for 7 min. Amplicons were analyzed on a 1.5% agarose gel and stained with ethidium bromide. Purification of PCR fragments for sequencing was performed by using a QIAquick PCR purification kit (Qiagen). For sequencing, the same primers as for PCR were applied, generating sequence data from both ends of amplicons. Assembly of DNA sequence files was conducted with the CAP3 Sequence Assembly Program (https://doua.prabi.fr/software/cap3 (accessed on 29 May 2024)) and primer sequences were clipped. Sequence identification was carried out by comparing them to NCBI GenBank entries (http://www.ncbi.nlm.nih.gov/blast/Blast.cgi (accessed on 29 May 2024)) using BLASTX [10]. The partial sequence of the 16S rDNA gene was deposited in GenBank under accession number PQ073124.

### 3.4. In Vivo Virulence Assay

*Galleria mellonella* larvae were bred in-house at an ambient atmosphere of 26 ± 1 °C in our Medical Entomology branch. The moth larvae were fed with daily portions of a diet consisting of the following stock ingredients: 250 g of bee honey, 250 g of glycerine, 100 g of dry yeast, 100 g of skim milk powder, 750 g of wheat bran, 250 g of oat flakes, and 20 g of the TetraMin^®^ fish diet (Tetra GmbH, Melle, Germany). Additionally, a few pellets of cera flava (yellow bee wax, Caesar & Loretz GmbH, Hilden, Germany) were separately added every other day. In order to attract gravid moths to lay eggs, we offered old honeycomb pieces sized 5 × 5 cm^2^. Consecutive populations were kept in plastic insectary boxes with a volume of approximately 6 L. Specimens with a weight > 0.3 g were injected with 10 μL of a bacterial solution of *P. straminea* or *P. aeruginosa* with a density of McFarland = 1, corresponding to a total number of 5 × 10^6^ colony-forming units. Serial ten-fold dilutions and undiluted volumes were assayed (*n* = 5–14). The *P. aeruginosa* strain ATCC 27853 was obtained from Axon Lab AG (Reichenbach, Germany) for quality control. For sham controls, larvae were injected with 10 μL of sterile PBS. Survival of the larvae was assayed every day for a week. Statistical analyses with the Mantel–Cox test and Kaplan–Meier survival curves were conducted with GraphPad Prism v8.4.3.

### 3.5. Scatter Electron Microscopy

For electron microscopic imaging, pure bacterial colonies were transferred from the solid culture into 4% buffered (0.1 mol cacodylate) Glutaraldehyde for 1 h at room temperature for fixation. Afterward, they were rinsed three times with a diluted buffer (0.2 mol). The specimens were then dehydrated with dilutions of alcohol, transferred into HMDS (Hexamethyldisilazane), brought in a suitable dilution holder, air-dried, sputtered with gold (Savematic CCU HV), and assessed with SEM (Gemini 360, Zeiss Medical technology, Ulm, Germany).

## 4. Results and Discussion

**Figure 3:**
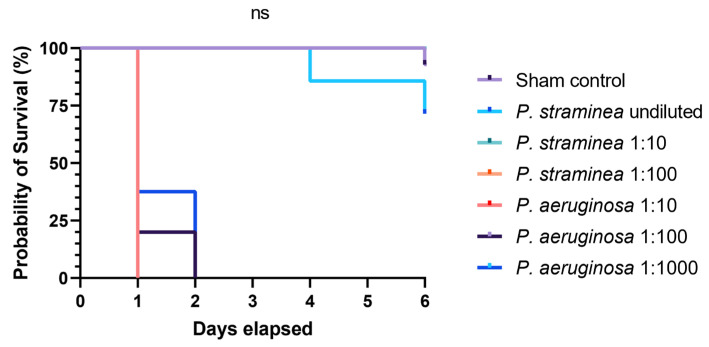
Kaplan–Meier survival curves of *Galleria mellonella* larvae following injections with either *P. straminea* or *P. aeruginosa*. *G. mellonella* larvae (*n* = 5–14) were injected including sterile PBS as sham controls. Survival of the larvae was observed for 6 days. GraphPad Prism was used for the statistical testing, applying a Mantel–Cox test for the Kaplan–Meier curve corresponding to the survival of sham controls compared to the curves of larvae injected with undiluted (5 × 10^6^ colony-forming units) volumes of *Pseudomonas* spp. The figure was created with GraphPad Prism v8.4.3. The green and orange *P. straminea* dilutions were equal to the purple sham line and are therefore not visible in the graph.

Here, we describe a bloodstream infection with *P. straminea* in an elderly woman that most likely developed from a skin and soft tissue infection (Figure 1). The patient suffered from a range of chronic diseases affecting the skin including allergies, atopic dermatitis, psoriasis, and chronic venous insufficiency, all of which are associated with an increased susceptibility to bacterial, viral, and fungal infections [11,12,13,14]. Other common causes of systemic infection such as pneumonia and urogenital infections were considered unlikely based on clinical examination and a lung X-ray taken at admission in the ED. The cellulitis was, therefore, considered the plausible cause for the symptoms that indicated a systemic infection. The putrid ulceration on the right hand (Figure 1b) was considered a likely point of entry because *P. straminea* was isolated from this site as well as from the bloodstream. After the isolation and identification of *P. straminea*, the antibiotic regimen was changed to ceftazidime, as recommended for the treatment of infection with *Pseudomonas* spp., followed by a swift and complete recovery of the patient concerning her infection. Follow-up blood cultures remained sterile and were performed to monitor restitution and rule out a biofilm-associated infection (e.g., in the CVC) and to differentiate between CVC infection and soft tissue infection. However, since they were taken during antimicrobial treatment with ceftazidime and no peripheral puncture was possible to compare peripheral and CVC blood culture time to positivity, they have to be interpreted with caution and only in the light of clinical restitution. The *Enterococcus* isolate was only isolated from the ulcer and was considered not clinically relevant, rather only colonizing the site. The patient fully recovered. Overall, *P. straminea* was reported from two independent sites. The clinical presentation of the ulcer alongside the therapeutic success of ceftazidime in contrast to clindamycin, which is not effective against *Pseudomonas* spp., makes an invasive *P. straminea* infection from a soft tissue focus the likely diagnosis. In contrast, a 16S rDNA sequencing-based analysis sorted *P. straminea* in a phylogenetic group with environmental *Pseudomonas* species that have not been reported to cause any infections. The apathogenic nature of *P. straminea* was underscored by the results from the *G. mellonella* infection model, a model used to investigate the virulence of bacteria [15]. In that model, we demonstrated the absence of any detrimental effects of injections with *P. straminea* with respect to the survival of the larvae. Almost all dilutions of *P. straminea* equaled the germ-free PBS injection in contrast to injections with *P. aeruginosa* (Figure 3). However, the patient provided susceptibility to a bacterial soft tissue infection with skin barrier dysfunctions caused by her chronic and severe skin diseases and immune system, which should be considered impaired [16]. The immunocompromised condition overall may facilitate the development of septicemia from a wound source. This case demonstrates how unusual bacteria that are considered non-pathogenic may cause serious complications in geriatric patients with chronic dermatologic diseases like atopic dermatitis and psoriasis. The correct identification of such pathogens may sometimes be difficult with standard microbiological techniques, as demonstrated here by the unsatisfying results from the VITEK-System. We, therefore, correlated our identification from clinical routine with a molecular analysis based on 16S rDNA gene sequencing. After PCR and clipping of primer sequences, in a 1426-base-pairs-long DNA fragment, a BLAST search against NCBI GenBank identified a 99.1% identity to *P. straminea* entries (accession numbers LC507441, LC507440, and LC420056). However, the number of reference genomes in such a rare species is relatively low and the method could still be too imprecise for definite discrimination, especially with respect to related species from the recently proposed *P. straminea* clade [8] or other *Pseudomonas* spp. that were described previously to have high similarity with *P. straminea* [17]. Two candidates from these studies (*Pseudomonas fulva* [17] and *P. flavescens* [8]) were also among the possible results from the BLAST search. However, the consistent *P. straminea* score from mass spectrometry with various repetitions from both isolation sites correlated with 16S rDNA sequencing. A recent study confirmed the good performance of MALDI-TOF with updated databases compared to 16S rDNA sequencing with respect to the identification at the species level in environmental *Pseudomonas* spp. [18]. Overall, the scarce data from environmental pathogens of low clinical importance complicate definite identification, and multiple techniques should be used for identification.

## 5. Conclusions

Immunosenescent patients affected by chronic skin diseases need to be aware of an increased risk of infectious complications even with bacteria of low virulence. Careful diagnostic approaches and microbiological results from different sites may prevent false interpretations of these environmental pathogens as contamination rather than relevant infectious agents. Identification at the species level may require molecular characterization alongside standard microbiological techniques.

## Figures and Tables

**Table 1:** Susceptibility of the *P. straminea* isolate.

Antimicrobial	MIC (mg/L)	Interpretation
Piperacillin	≤4	I
Piperacillin/Tazobactam	≤4	I
Ceftazidime	1	I
Cefepime	0.5	I
Aztreonam	16	I
Imipenem	≤0.5	I
Meropenem	≤0.25	S
Amikacin	≤1	S
Tobramycin	≤2	S
Ciprofloxacin	≤0.06	I

*P. straminea* antibiotic susceptibility based on the EUCAST breakpoints for *Pseudomonas* spp. (Version 13.00, valid from 1 January 2023). MIC = minimum inhibitory concentration, S = susceptible, standard dosing regimen, I = susceptible, increased exposure

## Data Availability

The original contributions presented in the report are included in the article and made accessible by accession number provided in the method section. Further inquiries can be directed to the corresponding author.
